# A cross-sectional study on anthropometric indices and hypertension in Chinese adults: comparison of discriminatory power and age-stratified analysis

**DOI:** 10.3389/fpubh.2026.1933929

**Published:** 2026-10-15

**Authors:** Shiqin Lu, Nan Chen, Zhuosong Li, Yuanmei Zhang, Wenping Wang, Guo Li, Jinglan Hao, Rui Wang, Yanmei Qi, Zhengyu Qin

**Affiliations:** 1 Health Management Center, The Affiliated Anning First People's Hospital of Kunming University of Science and Technology, Kunming, Yunnan, China; 2 School of Basic Medical Science, Kunming University of Science and Technology, Kunming, Yunnan, China; 3 Department of Endocrinology, The Affiliated Anning First People's Hospital of Kunming University of Science and Technology, Kunming, Yunnan, China; 4 Department of Scientific Research Management, The Affiliated Anning First People's Hospital of Kunming University of Science and Technology, Kunming, Yunnan, China; 5 Population-wide Health Management Center of Yongren County, Chuxiong, Yunnan, China; 6 Health Bureau of Yongren County, Chuxiong, Yunnan, China

**Keywords:** ABSI, BMI, BRI, WC, WHtR

## Abstract

**Background:**

Hypertension imposes a heavy public health burden in China. Simple anthropometric indicators help screen hypertension risk, yet the optimal index differs by age. This study compares five adiposity indices including WC, BMI, WHtR, ABSI, and BRI and their age-specific discriminatory ability among Chinese adults.

**Materials and methods:**

In this cross-sectional analysis of 104,248 adults from a health check-up cohort in Southwest China, hypertension was defined as blood pressure ≥140/90 mmHg, a self-reported diagnosis, or current antihypertensive medication use. Multivariable logistic and linear regression models were employed to examine the associations between a one-standard-deviation increase in the Z-score of each index and hypertension prevalence or blood pressure levels. Discriminatory power was compared using the area under the receiver operating characteristic curve (AUC) overall and within age groups.

**Results:**

Per standard deviation increase, WC, BMI, WHtR, ABSI, and BRI were associated with a 61% (OR 1.61, 95% CI: 1.58–1.64), 61% (1.61, 1.58–1.64), 60% (1.60, 1.57–1.63), 9% (1.09, 1.07–1.11), and 56% (1.56, 1.54–1.59) higher hypertension risk, respectively. WHtR and BRI showed the highest and identical AUC (both 0.676), followed by WC (0.653). ABSI had the lowest AUC (0.612). Discriminatory ability was strongest in adults aged 18–35 years, with WC performing best (AUC = 0.763). The associations for all indices significantly weakened with age (*P* for interaction < 0.001).

**Conclusions:**

WHtR and BRI demonstrated the strongest overall associations with hypertension, whereas WC was the best indicator in young adults. The performance of anthropometric indices varied by age, suggesting that age-specific considerations may enhance their utility for preliminary hypertension risk screening in practice.

## 1 Introduction

Hypertension remains one of the most prevalent and burdensome modifiable cardiovascular risk factors worldwide (1, 2). The prevalence of hypertension among Chinese adults remains persistently high. Despite recent improvements in awareness, treatment, and control rates, the overall task of prevention and management remains formidable (3, 4). Using simple and accessible anthropometric indicators to identify individuals at high risk of hypertension in clinical settings such as health check-ups and chronic disease management has clear practical utility.

The association between obesity and hypertension is well-established, with underlying mechanisms involving multiple pathways, including activation of the sympathetic nervous system and the renin-angiotensin-aldosterone system, renal sodium and water retention, inflammatory responses, and vascular dysfunction (5–7). In clinical practice, waist circumference (WC) is an established vital sign of visceral adiposity. Common anthropometric indices include body mass index (BMI), WC, waist-to-height ratio (WHtR), body roundness index (BRI), and a body shape index (ABSI), which provide complementary information: BMI reflects overall mass, WC and WHtR indicate abdominal fat, BRI captures body roundness, and ABSI adjusts WC for height and weight (9–11).

All anthropometric indices confirm an association with hypertension, though their relative performance differs within a given population. Systematic reviews and meta-analyses collectively suggest that WHtR, WC, and BRI generally outperform or are comparable to BMI for risk discrimination, whereas ABSI tends to demonstrate weaker performance (12, 13). In Chinese populations, a prospective study based on the China Health and Nutrition Survey (CHNS) suggests that BRI outperforms ABSI in identifying new-onset hypertension, although its discriminatory ability declines with longer follow-up duration (14).

Recent cross-sectional studies from the United States and China indicate that the relative performance of WHtR, BRI, and WC can vary with the age structure and characteristics of the population studied. Furthermore, the comparative advantage of BMI vs. WC in large Chinese cohorts may also differ by sex (15, 16). Therefore, current evidence does not support a single anthropometric index as universally optimal; its relative performance is context-dependent, influenced by population characteristics and the specific clinical or public health application.

Moreover, age may act as an effect modifier, not merely a confounder, influencing the association between adiposity and hypertension. Variations in this relationship across populations, as seen in studies from rural Yunnan and Taiwan, underscore the role of population characteristics and context (17, 18). To address this, we analyzed a health check-up cohort from Southwest China to compare five anthropometric indices (WC, BMI, WHtR, BRI, ABSI) in relation to hypertension and blood pressure, and to assess variations across age strata, thereby aiming to generate context-specific evidence for hypertension risk screening in clinical practice.

## 2 Materials and methods

### 2.1 Study participants and data source

This study was a retrospective cross-sectional analysis based on an existing hospital health examination database. The participants were adults who underwent health check-ups at the Health Management Center of Anning First People's Hospital, affiliated with Kunming University of Science and Technology, between January 2019 and December 2024. The database system integrates participants' physical measurements, laboratory test results, and questionnaire survey information. All extracted study data were de-identified and used solely for scientific research and analysis.

The study protocol was approved by the Hospital Ethics Committee (Approval No.: 2025-047 (Zi-Heng)-02), and a waiver of individual informed consent was granted.

### 2.2 Study design

This was a cross-sectional analysis of consecutive health examination records from the Affiliated Anning First People's Hospital, Kunming University of Science and Technology (January 2019 to December 2024). The initial database contained 214,582 records. Based on pre-defined criteria, records were sequentially excluded for missing data on key variables (*n* = 94,319), missing medical history (*n* = 13,025), age < 18 years (*n* = 2,033), outliers in anthropometric measures (*n* = 471), and duplication (*n* = 486). The final analytical sample comprised 104,248 participants. Duplicate records (retaining the earliest complete entry) and physiologically implausible values were removed per pre-specified quality-control rules. The participant selection process is detailed in Figure 1. Among the excluded records with available demographic data (*n* = 109,848), the mean age was 35.6 ± 16.4 years and 71.5% were male, indicating that excluded participants were generally younger and more predominantly male than in the included cohort (mean age 48.15 ± 14.72 years, 58.4% male). This difference suggests potential selection bias, as further discussed in the limitations section.

**Figure 1:**
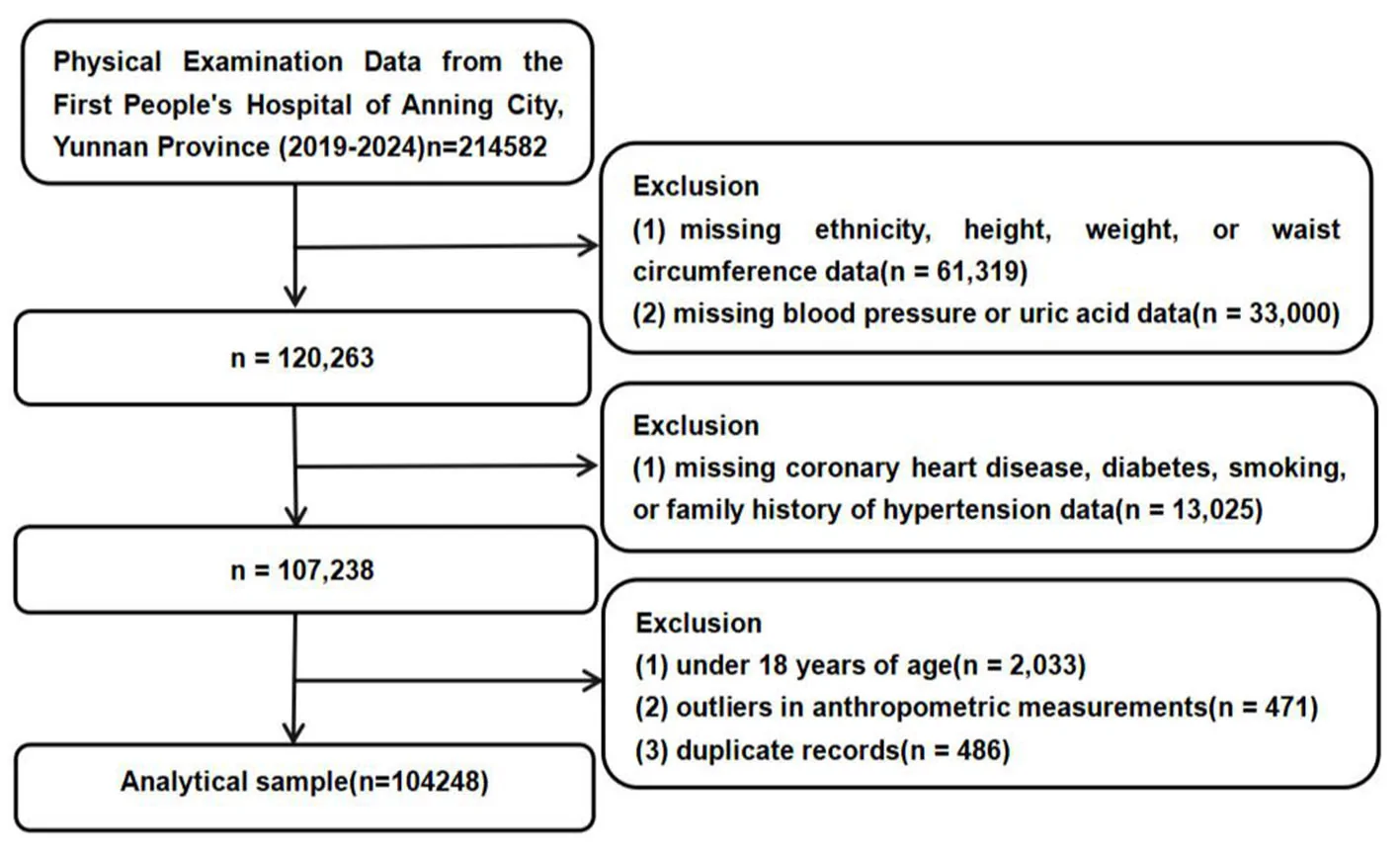
Flowchart of study population selection.

Anthropometric measurements (height, weight, waist circumference) were obtained following the health examination center's standardized protocols. Office blood pressure was measured in one upper arm using an electronic sphygmomanometer (Omron; the specific model is documented in the examination center's records). Appropriate cuff sizes were selected according to participants' upper-arm circumference. Participants remained in a seated position following a 5-min rest before measurement. For each participant, two readings were taken at a single visit with at least 1 min between readings, and the average of the two was used; if the difference between the two readings exceeded 5 mmHg, a third measurement was taken and the mean of the three was used. The device was regularly calibrated according to the manufacturer's instructions, and the zero error of the device was verified before each measurement session; all measurements were performed by trained nurses following a standardized protocol. Fasting venous blood samples were collected for the determination of serum uric acid levels. Body mass index (BMI), waist-to-height ratio (WHtR), a body shape index (ABSI), and body roundness index (BRI) were subsequently calculated using their respective standard formulas.


$$\begin{array}{l} \text{BMI}=\frac{\text{Weight}\left(\text{kg}\right)}{\text{Height}{\left(\text{m}\right)}^{2}}\\ \text{WHtR}=\frac{\text{Waist circumference}\left(\text{cm}\right)}{\text{Height}\left(\text{cm}\right)}\\ \text{ABSI}=\frac{\text{Waist circumference}\left(\text{m}\right)}{{\text{BMI}}^{2/3}\times \text{Height}{\left(\text{m}\right)}^{1/2}\;}\\ \text{BRI}=364.2-365.5\times \sqrt{1-\frac{{\left(\frac{\text{Waist circumference}\left(\text{cm}\right)}{2\pi }\right)}^{2}}{{\left(0.5\times \text{Height}\left(\text{m}\right)\right)}^{2}}} \end{array}$$

The diagnostic criteria for hypertension in this study referred to the definition in the Chinese Guidelines for the Management of Hypertension (2024 Revision). According to these guidelines, hypertension was defined as a systolic blood pressure (SBP) ≥140 mmHg and/or a diastolic blood pressure (DBP) ≥90 mmHg, a self-reported previous diagnosis of hypertension, or current use of antihypertensive medication (19).

The covariates extracted from the study database included: sex (male, female), age (18–97 years), ethnicity (Han, other ethnicities), smoking history (yes, no), family history of hypertension (yes, no), history of diabetes (yes, no), history of coronary heart disease or myocardial infarction (yes, no), and serum uric acid level (continuous variable, μmol/L). These variables were included for adjustment in the multivariable models based on their clinical relevance and data availability.

Ethnicity was categorized as Han or other. Age was stratified as young adults (18–35 years), middle-aged (36–59 years), and older adults (≥ 60 years), based on the sample distribution, clinical interpretability, and common age classifications in China, to evaluate age-specific differences in the associations between anthropometric indices and hypertension.

### 2.3 Statistical analysis

Analyses were conducted using SPSS 31.0 and Python 3.12. Continuous variables are expressed as mean ± SD and categorical variables as *n* (%). Baseline characteristics were compared by hypertension status using *t*-tests/Mann-Whitney U tests for continuous variables and chi-squared tests for categorical variables. To enable comparison, WC, BMI, WHtR, ABSI, and BRI were converted to Z-scores. Multivariable linear and logistic regression models assessed the associations of each index with blood pressure and hypertension. The discriminatory ability of each index was evaluated using the area under the receiver operating characteristic curve (AUC) and compared pairwise via the DeLong test, overall and by age stratum. Effect modification by age was tested by including an interaction term (age × index Z-score) in logistic models. A sensitivity analysis excluded participants on antihypertensive medication. All tests were two-sided, with *P* < 0.05 considered significant.

## 3 Results

### 3.1 Characteristics of the study population

After stepwise screening of 214,582 health examination records, 104,248 participants were ultimately included in the analysis (Figure 1). The exclusions sequentially comprised records missing data on ethnicity, height, weight, waist circumference, blood pressure, or uric acid (*n* = 94,319); records missing history of coronary heart disease, diabetes, smoking, or family history of hypertension (*n* = 13,025); participants aged < 18 years (*n* = 2,033) and those flagged as outliers (*n* = 471); and duplicate records (*n* = 486). The mean age of the final included population was 48.15 ± 14.72 years, 58.4% were male, and the prevalence of hypertension was 20.4% (*n* = 21,241). As shown in Table 1, compared to the normotensive group, participants in the hypertensive group were older, had significantly higher levels of all obesity indices (WC, BMI, WHtR, ABSI, BRI) and uric acid (*P* < 0.001), and had a higher proportion of males, individuals of Han ethnicity, and those with a family history of hypertension, a history of diabetes, and a history of coronary heart disease or myocardial infarction.

**Table 1:** Characteristics of the study population.

Hypertension	Negative	Positive	*P* ^*^	Total
Participants, *n*	83,007	21,241		104,248
Age (years), Mean ± SD	45.88 ± 14.20	57.01 ± 13.31	< 0.001	48.15 ± 14.72
WC (cm), Mean ± SD	81.84 ± 9.67	87.08 ± 9.31	< 0.001	82.91 ± 9.83
BMI (kg/m^2^), Mean ± SD	23.72 ± 3.32	25.24 ± 3.48	< 0.001	24.03 ± 3.41
WHtR, Mean ± SD	0.50 ± 0.06	0.53 ± 0.05	< 0.001	0.51 ± 0.06
ABSI, Mean ± SD	0.08 ± 0.01	0.08 ± 0.01	< 0.001	0.08 ± 0.01
BRI, Mean ± SD	3.39 ± 1.07	4.07 ± 1.11	< 0.001	3.53 ± 1.11
Uric acid (μmol/L), Mean ± SD	383.46 ± 100.46	407.05 ± 105.63	< 0.001	388.27 ± 101.98
Gender, *n* (%)			< 0.001	
Male	47,449 (57.2%)	13,464 (63.4%)		60,913 (58.4%)
Female	35,558 (42.8%)	7,777 (36.6%)		43,335 (41.6%)
Ethnicity, *n* (%)			< 0.001	
Han	69,961 (84.3%)	18,405 (86.6%)		88,366 (84.8%)
Minority	13,046 (15.7%)	2,836 (13.4%)		15,882 (15.2%)
Smoking, *n* (%)			< 0.001	
Non-smoking	59,001 (71.1%)	15,358 (72.3%)		74,359 (71.3%)
Smoking	24,006 (28.9%)	5,883 (27.7%)		29,889 (28.7%)
Family history of hypertension, *n* (%)			< 0.001	
No	24,269 (29.2%)	4,184 (19.7%)		28,453 (27.3%)
Yes	58,738 (70.8%)	17,057 (80.3%)		75,795 (72.7%)
Diabetes history, *n* (%)			< 0.001	
No	76,621 (92.3%)	18,831 (88.7%)		95,452 (91.6%)
Yes	6,386 (7.7%)	2,410 (11.3%)		8,796 (8.4%)
History of coronary heart disease/myocardial infarction, *n* (%)			< 0.001	
No	80,924 (97.5%)	20,535 (96.7%)		101,459 (97.3%)
Yes	2,083 (2.5%)	706 (3.3%)		2,789 (2.7%)

Data are presented as mean ± standard deviation (SD) for continuous variables and *n* (%) for categorical variables. ^*^P values were calculated by *t*-test for continuous variables and chi-square test for categorical variables. The total column presents combined data for the total study population (*n* = 104,248). WC, waist circumference; BMI, body mass index; WHtR, waist-to-height ratio; ABSI, a body shape index; BRI, body roundness index; SD, standard deviation.

### 3.2 Univariate analysis

In the univariate analysis (Table S1), age showed a significant positive association with hypertension. For each one-year increase in age, the risk of hypertension increased by approximately 6% (*P* < 0.001). Compared to males, females had a lower risk of hypertension (OR = 0.69, 95% CI: 0.64–0.76), and both systolic and diastolic blood pressure levels were relatively lower.

Regarding personal and family medical history, participants with a family history of hypertension had a 1.62-fold higher risk of the disease compared to those without such a history (OR = 1.62, 95% CI: 1.57–1.69). A history of diabetes and a history of coronary heart disease or myocardial infarction were also associated with a higher risk of hypertension (OR = 1.58, 95% CI: 1.50–1.66; OR =1.36, 95% CI: 1.24–1.48, respectively).

Among the five anthropometric indices, the Z-score of BMI showed the strongest univariate association with the risk of hypertension (OR = 2.41, 95% CI: 2.33–2.49). WHtR and BRI exhibited greater magnitudes of association with increased systolic blood pressure, suggesting a potentially closer relationship between central obesity indices and elevated blood pressure.

### 3.3 Trend analysis

To assess the robustness of the associations, each anthropometric index was categorized into quartiles (Supplementary Table S2). The associations with blood pressure and hypertension were examined using progressively adjusted models: unadjusted (Model I), adjusted for age, sex, and ethnicity (Model II), and further adjusted for smoking, family history of hypertension, diabetes, and coronary heart disease (Model III). A consistent, positive dose-response relationship was observed across all quartile models, reinforcing the stability of the primary findings.

### 3.4 Stratified analysis

Following stratification by gender (Supplementary Table S3), in the fully adjusted model, ABSI was not significantly associated with diastolic blood pressure in women (β = −0.05, *P* = 0.320), while WC, BMI, WHtR, and BRI remained significantly and positively associated in both men and women. The strength of association between most indices and hypertension was slightly higher in men than in women.

After stratification by ethnicity (Supplementary Table S4), all five indices were significantly associated with the risk of hypertension and blood pressure levels in both the Han and other ethnic groups. The strength of association for each index was generally similar across ethnicities, and no clear ethnic differences were observed.

In the age-stratified analysis (Table 2), the strength of association for all indices with hypertension (odds ratios) progressively declined with age, from the young adult group (18–35 years) to the older adult group (≥60 years). For systolic and diastolic blood pressure (linear regression coefficients), the associations were generally strongest in the middle-aged group (36–59 years), followed by the young adult group, and weakest in the older adults (≥60 years), indicating that the relationship between adiposity and blood pressure levels is not strictly linear across age. For example, in the fully adjusted model, the odds ratios (ORs) for hypertension associated with WHtR were 2.31, 1.77, and 1.31 in the young adult, middle-aged, and older adult groups, respectively. The association between ABSI and diastolic blood pressure was weakly inverse in the older adult group (β = −0.44).

**Table 2:** Stratified analysis by age.

Exposure	Age (years) = < 36	Age (years) = ≥36, < 60	Age (years) = ≥60	Total
Hypertension (OR)				
WC (cm) Z score	2.19 (2.06, 2.33)	1.73 (1.69, 1.77)	1.29 (1.25, 1.33)	1.76 (1.73, 1.79)
	< 0.001	< 0.001	< 0.001	< 0.001
BMI (kg/m^2^) Z score	2.01 (1.90, 2.12)	1.65 (1.61, 1.68)	1.27 (1.23, 1.31)	1.51 (1.49, 1.54)
	< 0.001	< 0.001	< 0.001	< 0.001
WHtR Z score	2.31 (2.17, 2.46)	1.77 (1.73, 1.81)	1.31 (1.27, 1.34)	1.88 (1.85, 1.91)
	< 0.001	< 0.001	< 0.001	< 0.001
ABSI Z score	1.27 (1.18, 1.37)	1.19 (1.17, 1.22)	1.10 (1.07, 1.12)	1.48 (1.46, 1.51)
	< 0.001	< 0.001	< 0.001	< 0.001
BRI Z score	2.23 (2.10, 2.36)	1.73 (1.69, 1.77)	1.29 (1.25, 1.32)	1.82 (1.79, 1.85)
	< 0.001	< 0.001	< 0.001	< 0.001
Mean systolic blood pressure (β)				
WC (cm) Z score	3.67 (3.52, 3.82)	4.81 (4.67, 4.94)	2.76 (2.51, 3.01)	5.29 (5.19, 5.40)
	< 0.001	< 0.001	< 0.001	< 0.001
BMI (kg/m^2^) Z score	3.64 (3.50, 3.77)	4.61 (4.49, 4.74)	2.64 (2.40, 2.88)	4.31 (4.21, 4.41)
	< 0.001	< 0.001	< 0.001	< 0.001
WHtR Z score	3.54 (3.39, 3.69)	4.92 (4.79, 5.05)	2.90 (2.67, 3.13)	5.66 (5.57, 5.75)
	< 0.001	< 0.001	< 0.001	< 0.001
ABSI Z score	0.21 (0.05, 0.38)	1.25 (1.11, 1.40)	0.94 (0.72, 1.17)	3.18 (3.08, 3.28)
	0.013	< 0.001	< 0.001	< 0.001
BRI Z score	3.72 (3.57, 3.88)	4.88 (4.75, 5.01)	2.73 (2.51, 2.95)	5.61 (5.51, 5.70)
	< 0.001	< 0.001	< 0.001	< 0.001
Mean diastolic blood pressure (β)				
WC (cm) Z score	2.24 (2.12, 2.35)	3.00 (2.90, 3.10)	1.13 (0.97, 1.28)	2.76 (2.69, 2.83)
	< 0.001	< 0.001	< 0.001	< 0.001
BMI (kg/m^2^) Z score	2.07 (1.97, 2.17)	2.81 (2.72, 2.90)	1.40 (1.26, 1.55)	2.54 (2.47, 2.60)
	< 0.001	< 0.001	< 0.001	< 0.001
WHtR Z score	2.20 (2.09, 2.32)	2.94 (2.85, 3.04)	0.70 (0.56, 0.84)	2.61 (2.55, 2.68)
	< 0.001	< 0.001	< 0.001	< 0.001
ABSI Z score	0.51 (0.38, 0.64)	0.80 (0.70, 0.90)	−0.44 (-0.57,−0.30)	0.94 (0.88, 1.01)
	< 0.001	< 0.001	< 0.001	< 0.001
BRI Z score	2.36 (2.24, 2.48)	2.93 (2.84, 3.02)	0.65 (0.51, 0.79)	2.58 (2.51, 2.64)
	< 0.001	< 0.001	< 0.001	< 0.001

Data in the table: β (95%CI) *p*-value/OR (95% CI) *p*-value. Outcome variables: Hypertension; Mean systolic blood pressure (mmHg); Mean diastolic blood pressure (mmHg). Exposure variables: ABSI Z score; WC (cm) Z score; BMI (kg/m^2^) Z score; WHtR Z score; BRI Z score. Model adjusted for: Gender; Race; Smoking status; Family history of hypertension; History of diabetes; History of CHD/MI. OR, odds ratio; CI, confidence interval; WC, waist circumference; BMI, body mass index; WHtR, waist-to-height ratio; ABSI, a body shape index; BRI, body roundness index; CHD, coronary heart disease; MI, myocardial infarction.

These findings indicate that the association between anthropometric indices and hypertension varies by age, and that age influences the relative performance of different indices. ABSI consistently showed the weakest association across all age strata.

### 3.5 Interaction analysis

To examine whether the patterns observed in the age-stratified analysis were statistically significant, formal tests for interaction were performed (Supplementary Table S5). The results showed that the interaction terms between age and all five anthropometric indices (WC, BMI, WHtR, ABSI, BRI) were highly statistically significant (all *P*-values < 0.001). All interaction term coefficients were negative (WHtR: β = −0.0147; WC: β = −0.0130; BRI: β = −0.0141; ABSI: β = −0.0091; BMI: β = −0.0075), indicating that the strength of the positive association between each index and hypertension systematically attenuated with increasing age.

### 3.6 Multivariable regression analysis

Based on the trend and stratified analyses, we further employed multivariable regression models to assess the independent effects of each anthropometric index in the overall sample (Supplementary Table S6). Model I was unadjusted; Model II was adjusted for sex, age, and ethnicity; Model III was further adjusted for smoking history, family history of hypertension, history of diabetes, history of coronary heart disease or myocardial infarction, and serum uric acid level.

After adjusting for all confounding factors, in the total sample, each one-standard-deviation increase in WC, BMI, WHtR, ABSI, and BRI was associated with a 61% (95% CI: 58%−64%), 61% (95% CI: 58%−64%), 60% (95% CI: 57%−63%), 9% (95% CI: 7%−11%), and 56% (95% CI: 54%−59%) increased risk of hypertension, respectively.

Similarly, for each one-standard-deviation increase in the aforementioned indices, systolic blood pressure (SBP) increased by 3.99 mmHg (95% CI: 3.89–4.09), 4.00 mmHg (95% CI: 3.91– 4.09), 3.95 mmHg (95% CI: 3.86–4.05), 0.61 mmHg (95% CI: 0.51–0.71), and 3.93 mmHg (95% CI: 3.83–4.02), respectively; diastolic blood pressure (DBP) increased by 2.47 mmHg (95% CI: 2.41–2.54), 2.46 mmHg (95% CI: 2.39–2.52), 2.28 mmHg (95% CI: 2.22–2.35), 0.27 mmHg (95% CI: 0.20–0.34), and 2.25 mmHg (95% CI: 2.18–2.31), respectively. The overall trend was consistent with the results from the logistic regression analysis, with ABSI showing the weakest effect.

### 3.7 ROC curve analysis

ROC curve analysis was further employed to evaluate the relative discriminatory ability of different anthropometric indices in identifying hypertension. In the overall population (*n* = 104,248) (Figure 2), the waist-to-height ratio (WHtR) and the body roundness index (BRI) showed the highest and identical discriminatory performance (both AUC = 0.676, 95% CI: 0.672–0.681). Waist circumference (WC) demonstrated the next best performance (AUC = 0.653, 95% CI: 0.649–0.657), followed by body mass index (BMI) (AUC = 0.627, 95% CI: 0.623–0.632), while the body shape index (ABSI) had the lowest discriminatory value (AUC = 0.612, 95% CI: 0.608–0.616). The DeLong test indicated (Table 3) that the AUC of WHtR was significantly higher than that of WC (difference = 0.023, *P* < 0.001), BMI (difference = 0.049, *P* < 0.001), and ABSI (difference = 0.064, *P* < 0.001), but its difference from BRI was not statistically significant (difference = 0, *P* = 1.000). Furthermore, the discriminatory performance of WC was also significantly superior to that of BMI and ABSI (both *P* < 0.001).

**Figure 2:**
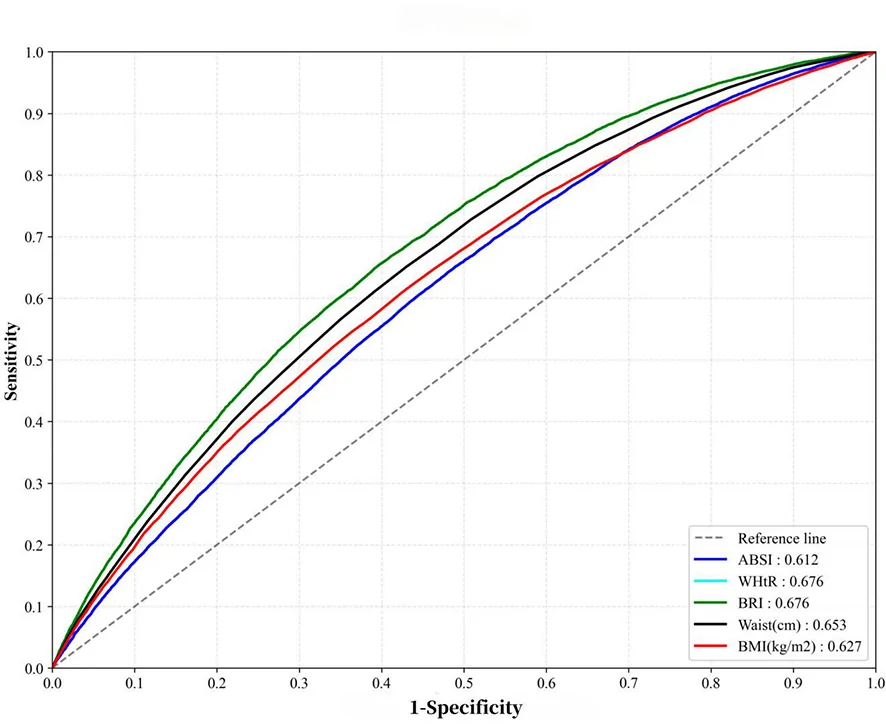
Receiver operating characteristic (ROC) curves for discriminating hypertension in the overall population. Detailed results of univariate, multivariable, and stratified analyses are provided in the (Supplementary Tables S1-S6).

**Table 3:** Pairwise comparisons of area under the receiver operating characteristic curve (AUC) between anthropometric indices, using the DeLong test.

Comparison	AUC_A	AUC_B	AUC difference	*P*-value
WHtR vs. ABSI	0.676	0.612	0.064	< 0.001
WHtR vs. BMI	0.676	0.627	0.049	< 0.001
WHtR vs. WC	0.676	0.653	0.023	< 0.001
WHtR vs. BRI	0.676	0.676	0.000	1.000
BRI vs. ABSI	0.676	0.612	0.064	< 0.001
BRI vs. BMI	0.676	0.627	0.049	< 0.001
BRI vs. WC	0.676	0.653	0.023	< 0.001
WC vs. BMI	0.653	0.627	0.026	< 0.001
WC vs. ABSI	0.653	0.612	0.041	< 0.001
BMI vs. ABSI	0.627	0.612	0.015	< 0.001

AUC, area under the receiver operating characteristic curve; WHtR, waist-to-height ratio; BRI, body roundness index; WC, waist circumference; BMI, body mass index; ABSI, a body shape index. *P* values were derived from the DeLong test for comparing two correlated AUCs. The performance difference between WHtR and BRI was not statistically significant.

Following age stratification (Figure 3), the relative ranking of the indices was not constant. In the young adult group (18–35 years), the AUCs for all indices were generally high, with WC demonstrating the best performance (AUC = 0.763, 95% CI: 0.753–0.773), followed closely by WHtR and BRI (AUC both 0.754, 95% CI: 0.744–0.764), suggesting that information related to central obesity may have higher discriminatory value in young adults. Upon entering the middle-aged and older adult groups, the AUCs of all indices gradually declined, and the differences between them noticeably diminished.

**Figure 3:**
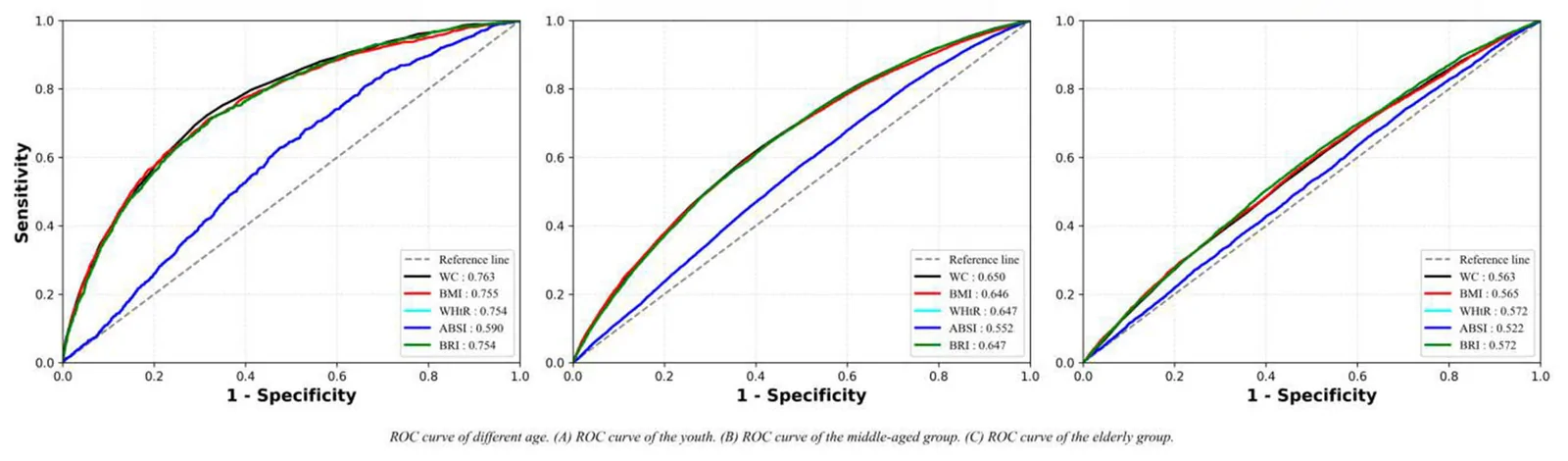
ROC curves for discriminating hypertension, stratified by age. **(A)** Youth group, **(B)** Middle-aged group, **(C)** Older adult group.

Further subgroup analyses within the young adult group (Figures 4, 5) showed that the aforementioned pattern remained largely stable. Across different ethnic subgroups, the AUC values for WC, WHtR, and BRI were approximately 0.75–0.77, all of which were higher than those for BMI and ABSI. After stratification by gender, the AUCs for all indices were generally slightly higher in young adult males than in young adult females, while ABSI demonstrated the lowest discriminatory ability in both groups.

**Figure 4:**
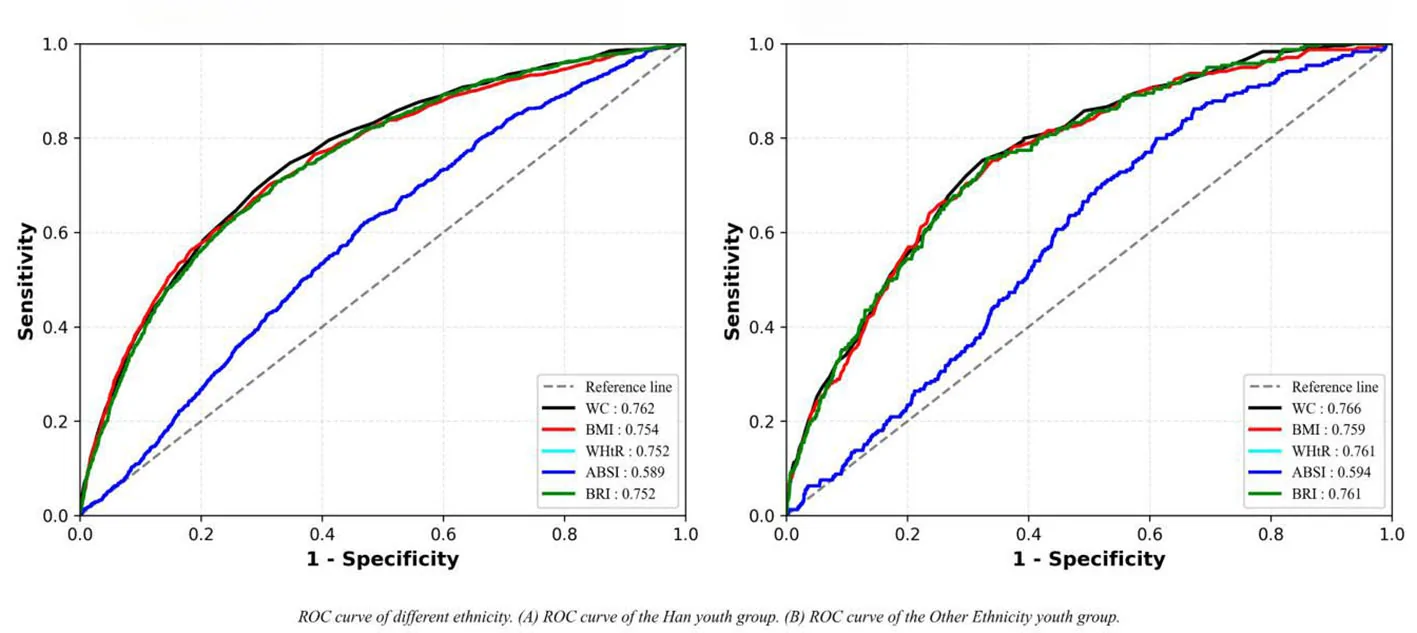
ROC curves for discriminating hypertension in the youth population, stratified by ethnicity. **(A)** Han youth group, **(B)** Other Ethnicity youth group.

**Figure 5:**
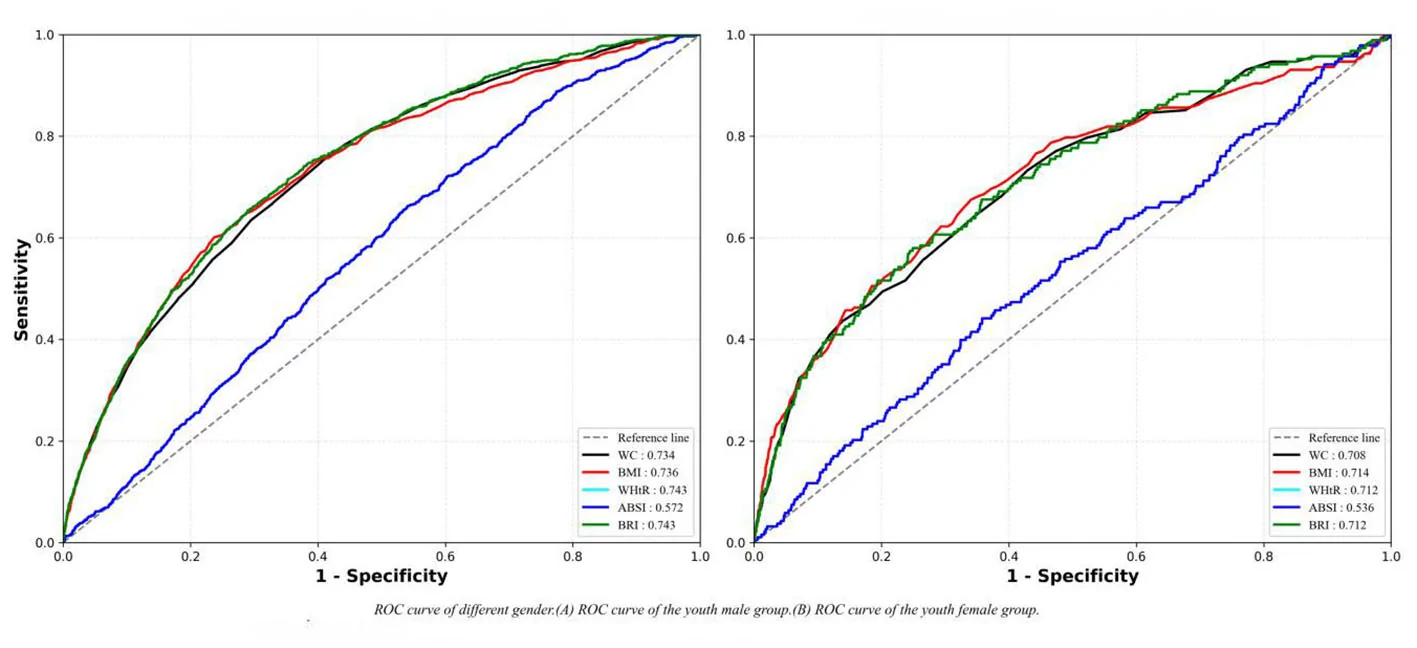
ROC curves for discriminating hypertension in the youth population, stratified by sex. **(A)** Youth male group, **(B)** Youth female group.

Overall, the performance ranking of anthropometric indices for identifying hypertension was context-dependent and varied across age strata. In the overall population, WHtR, BRI, and WC showed comparable performance, all superior to ABSI. Within the young adult group, WC demonstrated a relatively higher discriminatory ability, a finding of interpretive value.

### 3.8 Sensitivity analysis

To evaluate the robustness of the main conclusions, a sensitivity analysis was conducted by excluding 1,463 participants who self-reported current use of antihypertensive medication from the original cohort, resulting in a sensitivity analysis cohort (SA1, *n* = 102,785). After repeating the multivariable logistic regression and ROC analyses in this cohort, the primary results showed no substantial change: the direction and strength ranking of the associations between each index and hypertension remained consistent, and WHtR and BRI still showed the highest and similar AUC values. This indicates that the main analysis results are robust.

## 4 Discussion

All five anthropometric indices were associated with hypertension and blood pressure, but their discriminatory ability varied. In the overall sample, WHtR and BRI demonstrated significantly superior discriminatory performance to BMI, while ABSI was consistently weaker, providing a statistical rationale for prioritizing central obesity measures. This aligns with previous systematic reviews and large-scale studies indicating that central obesity indices often perform as well as or better than BMI, though with generally modest discriminatory accuracy (12, 15, 16). Therefore, from a clinical application perspective, these indices are more suitable for preliminary risk identification in settings such as health check-ups or primary care, and should not be interpreted as standalone diagnostic criteria or comprehensive discriminatory models.

It should be noted that BRI and WHtR are mathematically near-equivalent in this context: BRI is essentially a monotonic transformation of the ratio of waist circumference to height (specifically, a function of WC divided by π times height, which is effectively the inverse of WHtR), rather than an independent measure. This explains their nearly identical effect estimates and AUC values in our data. The observed AUC values were identical for both indices (both 0.676, DeLong *P* = 1.000), further confirming their mathematical near-equivalence. Therefore, our finding that both indices perform similarly does not imply that they capture distinct biological information; rather, they both reflect the same underlying central obesity signal. We interpret them as two variants of the same metric, and the choice between them in practice may depend on computational simplicity (WHtR) or theoretical appeal (BRI).

These results suggest that a simple comparison of the numerical values of different indices is insufficient to summarize their clinical significance. The development of obesity-related hypertension involves multiple mechanisms, including neurohumoral activation, renal sodium and water retention, inflammation, and vascular dysfunction, with abdominal/visceral fat distribution potentially playing a more direct role (5, 7, 8). Correspondingly, WC, WHtR, and BRI place greater emphasis on abdominal fat load or body roundness information, and have often demonstrated relatively stable discriminatory ability in studies across different populations (15, 16, 20). Waist circumference itself has also been proposed as an important component of routine clinical assessment (6). However, this relative advantage cannot be viewed as evidence for a universal optimal index, as studies differ in age structure, ethnicity, covariate adjustment, and outcome definitions (16, 21–23).

A key finding was the altered performance ranking of indices after age stratification. Waist circumference (WC), waist-to-height ratio (WHtR), and body roundness index (BRI) demonstrated superior discriminatory ability in young adults (18–35 years), while discriminatory power declined and differences between indices converged in older adults—a pattern consistent with other Asian cohorts. Our interaction analysis provides the first statistical evidence that age significantly attenuates the association for all five indices (all *P* for interaction < 0.001), confirming a broad “age-attenuation effect.” This suggests that with advancing age, blood pressure is increasingly determined by multifactorial determinants (e.g., vascular changes, comorbidities), which may diminish the standalone discriminatory value of anthropometric indices, consistent with findings in other Chinese populations (17, 24). This mechanism also reasonably explains why central obesity indices like WC and WHtR achieved the highest AUCs in the young adult group, as obesity, being the dominant risk factor at this stage, exerts its strongest signal, unattenuated by other age-related comorbidities. A plausible mechanistic explanation for the superior performance of raw WC over height-normalized indices in young adults is that in this age group, height variability is relatively small whereas waist circumference exhibits greater inter-individual variation driven by lifestyle and dietary habits; normalizing WC by height (as in WHtR and BRI) may therefore introduce noise rather than signal, slightly reducing discriminative ability.

The results for ABSI provide a contrasting reference to the above interpretation. This index was originally designed to characterize body shape differences more finely by adjusting waist circumference for height and BMI (10). However, in this study, ABSI showed weaker associations with hypertension and blood pressure and consistently lower discriminatory ability (AUC) compared to other indices. This aligns with previous evidence, including meta-analyses and studies in US and Chinese populations (general, diabetic, and prehypertensive adults), which generally report that ABSI underperforms relative to WC, WHtR, or BRI for identifying hypertension-related risk (12, 15, 25–27). This suggests that greater mathematical sophistication does not necessarily translate into superior clinical identification capability.

This study also underscores that the comparative utility of anthropometric indices is context-specific. Although central obesity-related indices have demonstrated value for risk identification in large Chinese cross-sectional studies, their optimal ranking varies across different study types, including those on Chinese adult obesity, prospective cohorts in Southwest China, and research on cardiometabolic comorbidities (16, 28–30). The interpretation of our results is limited to the cross-sectional, health screening setting. Despite strengths like a large sample and direct comparison of multiple indices, several limitations warrant caution: First, the exclusion of a substantial number of records (approximately 50%) due to missing data may introduce selection bias. In health check-up databases, missingness is often non-random; among excluded records with available demographic information (*n* = 109,848), the mean age was 35.6 ± 16.4 years and 71.5% were male, compared with 48.15 ± 14.72 years and 58.4% male in the included cohort. The younger age and male predominance among excluded records indicate that missingness was not completely random. Because excluded individuals were younger and likely at lower baseline cardiovascular risk, the complete-case analysis may have slightly overestimated the true population-level AUCs and association strengths, as the included cohort was enriched for older, higher-risk individuals. Specifically, the complete-case selection may have inflated the observed AUCs by preferentially retaining older participants with more complete records and higher baseline risk, which could strengthen the apparent adiposity–hypertension association. Second, the observed attenuation of associations in the in older adults could partly reflect survivorship bias: obese individuals with hypertension face significantly higher mortality rates, meaning they are systematically less likely to be alive at age 60+ and attend health check-ups. Consequently, the older cohort in the study represents a selected, disproportionately healthy sample, which would artificially attenuate the observed obesity–hypertension relationship relative to the general population. This competing explanation should be considered alongside the age-related physiological changes discussed. Third, we lacked data on several important lifestyle factors, including alcohol consumption, dietary sodium intake, physical activity, and sleep duration, all of which are known to influence blood pressure. Residual confounding from these unmeasured variables cannot be excluded and may have affected the strength and ranking of the associations. Finally, the cross-sectional design precludes causal inference; and the single-center data limit generalizability. Consequently, the observed ranking is indicative for similar settings. External validation in prospective, diverse cohorts is essential to assess general applicability.

## 5 Conclusion

This study shows that, within this single-center health check-up cohort of Chinese adults, the discriminatory ability for hypertension varied among different anthropometric indices. Overall, central obesity indices—represented by WHtR and BRI (two mathematically near-equivalent variants of the same waist-to-height construct)—demonstrated relatively superior discriminatory performance, while WC showed the highest discriminatory power in the young adult population. This ranking differed across age strata, suggesting that age may influence the relative performance of these indices. This age-specific advantage of raw WC is explored mechanistically in the Discussion, where we note that height varies little while waist circumference varies widely among young adults, so that height normalization can add noise rather than signal. Our findings have practical implications for clinical screening. In health check-up settings, using WC (especially in young adults) or WHtR/BRI (across all ages) can help identify individuals at higher hypertension risk. Once flagged, these individuals should undergo confirmatory blood pressure measurements and be counseled on lifestyle modifications, including weight management, dietary sodium reduction, and increased physical activity. Recent evidence suggests that interventions targeting adiposity-related risk captured by these indices can translate into measurable blood pressure benefits (31). Therefore, these simple anthropometric measures serve not only as screening tools but also as actionable targets for preventive strategies. In summary, central obesity indices could be utilized for the preliminary identification of individuals at high risk for hypertension in health check-up and primary care settings; however, their application value still requires validation in further research, taking into account specific population characteristics.

## Data Availability

The original contributions presented in the study are included in the article/Supplementary material, further inquiries can be directed to the corresponding authors.
