# Supplementary material for: A cross-sectional study on anthropometric indices and hypertension in Chinese adults: comparison of discriminatory power and age-stratified analysis
Source: Front Public Health. 2026 Oct 15;14:1933929. doi: 10.3389/fpubh.2026.1933929 (PMC13716726; doi:10.3389/fpubh.2026.1933929)
Supplement: Supplementary file 1 [file Data_Sheet_1.docx]

**Table S1**

**Univariate Regression Analysis of Hypertension and Blood Pressure**

| **Variables** | **Statistics (Mean±SD/n(%))** | **Hypertension (OR, 95%CI, P)** | **Systolic BP (β, 95%CI, P)** | **Diastolic BP (β, 95%CI, P)** |
| --- | --- | --- | --- | --- |
| Age (years) | 48.15 ± 14.72 | 1.06 (1.05, 1.06) | 0.45(0.44,0.45) <0.001 | 0.11(0.11,0.12) <0.001 |
| Waist Z-score | 0.00 ± 1.00 | 1.77 (1.74, 1.79) <0.001 | 5.66 (5.57, 5.75) <0.001 | 3.11 (3.05, 3.17) <0.001 |
| BMI Z-score | 0.00 ± 1.00 | 2.41 (2.33, 2.49) <0.001 | 2.26 (2.16, 2.36) <0.001 | 1.35 (1.29, 1.42) <0.001 |
| WHtR Z-score | 0.00 ± 1.00 | 1.93 (1.90, 1.96) <0.001 | 5.91 (5.82, 6.01) <0.001 | 2.72 (2.66, 2.78) <0.001 |
| ABSI Z-score | 0.00 ± 1.00 | 1.59 (1.56, 1.62) <0.001 | 2.86 (2.76, 2.96) <0.001 | 0.93 (0.86, 0.99) <0.001 |
| BRI Z-score | 0.00 ± 1.00 | 1.84 (1.81, 1.87) <0.001 | 5.96 (5.86, 6.05) <0.001 | 2.71 (2.65, 2.78) <0.001 |
| Uric Acid Z-score | 0.00 ± 1.00 | 1.25 (1.23, 1.26) <0.001 | 2.36 (2.26, 2.46) <0.001 | 1.81 (1.75, 1.87) <0.001 |
| Gender |  |  |  |  |
| Male (Ref.) | 60,913 (58.4%) | 1.0 | 0 | 0 |
| Female | 43,335 (41.6%) | 0.69 (0.64, 0.76) <0.001 | -5.77 (-6.20, -5.35) <0.001 | -2.80 (-3.10, -2.50) <0.001 |
| Ethnicity |  |  |  |  |
| Han | 88,366 (84.8%) | 1.22 (1.17, 1.27) <0.001 | 1.62 (1.34, 1.90) <0.001 | 0.28 (0.10, 0.46) 0.003 |
| Other (Ref.) | 15,882 (15.2%) | 1.0 | 0 | 0 |
| Smoking Status |  |  |  |  |
| Non-smoker (Ref.) | 74,359 (71.3%) | 1.0 | 0 | 0 |
| Smoker | 29,889 (28.7%) | 0.97 (0.93, 1.00) 0.042 | 0.25 (0.03, 0.48) 0.026 | 1.88 (1.74, 2.02) <0.001 |
| Hypertension Family History |  |  |  |  |
| No (Ref.) | 28,453 (27.3%) | 1.0 | 0 | 0 |
| Yes | 75,795 (72.7%) | 1.62 (1.57, 1.69) <0.001 | 2.16 (1.93, 2.38) <0.001 | 1.93 (1.79, 2.08) <0.001 |
| Diabetes History |  |  |  |  |
| No (Ref.) | 95,452 (91.6%) | 1.0 | 0 | 0 |
| Yes | 8,796 (8.4%) | 1.58 (1.50, 1.66) <0.001 | 5.48 (5.12, 5.84) <0.001 | 1.04 (0.80, 1.27) <0.001 |
| CHD/MI History |  |  |  |  |
| No (Ref.) | 101,459 (97.3%) | 1.0 | 0 | 0 |
| Yes | 2,789 (2.7%) | 1.36 (1.24, 1.48) <0.001 | 4.16 (3.53, 4.78) <0.001 | 0.54 (0.13, 0.94) 0.010 |

Abbreviations: OR = Odds Ratio; CI = Confidence Interval; BMI = Body Mass Index; WHtR = Waist-to-Height Ratio; ABSI = A Body Shape Index; BRI = Body Roundness Index; BP = Blood Pressure.
Reference groups: "Ref." indicates the reference group for categorical variables: Male (Gender); Other (Ethnicity); No (Diabetes History/CHD/MI History/Hypertension Family History); Non-smoker (Smoking Status).
Data presentation: Continuous variables (Age, Waist Z-score, etc.) are expressed as Mean±SD; categorical variables (Gender, Ethnicity, etc.) are expressed as n(%).
Statistical models: Logistic regression was used for the binary outcome "Hypertension" (expressed as OR, 95%CI, P); linear regression was used for continuous outcomes "Systolic BP" and "Diastolic BP" (expressed as β, 95%CI, P).
Statistical significance: All tests were two-tailed, with a significance level of α=0.05 (P<0.05 was considered statistically significant).

| **Table S2**  **Trend analysis** | | | |
| --- | --- | --- | --- |
| **Exposure** | **Model I** | **Model II** | **Model III** |
| **Mean systolic blood pressure (mmHg) (β)** | | | |
| **WC (cm)** |  |  |  |
| Q1 | 0.00 (Ref.) | 0.00 (Ref.) | 0.00 (Ref.) |
| Q2 | 6.17 (5.91, 6.44) <0.001 | 3.69 (3.44, 3.95) <0.001 | 3.74 (3.49, 4.00) <0.001 |
| Q3 | 10.01 (9.75, 10.28) <0.001 | 6.40 (6.13, 6.66) <0.001 | 6.44 (6.18, 6.70) <0.001 |
| Q4 | 13.95 (13.67, 14.23) <0.001 | 10.23 (9.95, 10.51) <0.001 | 10.25 (9.97, 10.53) <0.001 |
| *P for trend* | *p < 0.001* | *p < 0.001* | *p < 0.001* |
| **BMI (kg/m2)** |  |  |  |
| Q1 | 0.00 (Ref.) | 0.00 (Ref.) | 0.00 (Ref.) |
| Q2 | 4.62 (4.35, 4.89) <0.001 | 2.91 (2.66, 3.17) <0.001 | 2.85 (2.60, 3.10) <0.001 |
| Q3 | 8.12 (7.84, 8.39) <0.001 | 5.77 (5.52, 6.03) <0.001 | 5.69 (5.44, 5.95) <0.001 |
| Q4 | 12.12 (11.85, 12.40) <0.001 | 10.27 (10.02, 10.53) <0.001 | 10.15 (9.89, 10.41) <0.001 |
| *P for trend* | *p < 0.001* | *p < 0.001* | *p < 0.001* |
| **WHtR** |  |  |  |
| Q1 | 0.00 (Ref.) | 0.00 (Ref.) | 0.00 (Ref.) |
| Q2 | 5.99 (5.72, 6.26) <0.001 | 3.26 (3.00, 3.52) <0.001 | 3.33 (3.07, 3.58) <0.001 |
| Q3 | 10.05 (9.78, 10.31) <0.001 | 5.99 (5.73, 6.25) <0.001 | 6.09 (5.83, 6.35) <0.001 |
| Q4 | 15.27 (15.00, 15.54) <0.001 | 10.07 (9.80, 10.33) <0.001 | 10.14 (9.88, 10.41) <0.001 |
| *P for trend* | *p < 0.001* | *p < 0.001* | *p < 0.001* |
| **ABSI** |  |  |  |
| Q1 | 0.00 (Ref.) | 0.00 (Ref.) | 0.00 (Ref.) |
| Q2 | 4.25 (3.97, 4.53) <0.001 | 1.17 (0.91, 1.44) <0.001 | 1.32 (1.05, 1.58) <0.001 |
| Q3 | 6.46 (6.18, 6.74) <0.001 | 1.46 (1.19, 1.74) <0.001 | 1.70 (1.43, 1.98) <0.001 |
| Q4 | 8.90 (8.62, 9.18) <0.001 | 1.33 (1.05, 1.61) <0.001 | 1.65 (1.37, 1.93) <0.001 |
| *P for trend* | *p < 0.001* | *p < 0.001* | *p < 0.001* |
| **BRI** |  |  |  |
| Q1 | 0.00 (Ref.) | 0.00 (Ref.) | 0.00 (Ref.) |
| Q2 | 5.99 (5.72, 6.26) <0.001 | 3.26 (3.00, 3.52) <0.001 | 3.33 (3.07, 3.58) <0.001 |
| Q3 | 10.05 (9.78, 10.31) <0.001 | 5.99 (5.73, 6.25) <0.001 | 6.09 (5.83, 6.35) <0.001 |
| Q4 | 15.27 (15.00, 15.54) <0.001 | 10.07 (9.80, 10.33) <0.001 | 10.14 (9.88, 10.41) <0.001 |
| *P for trend* | *p < 0.001* | *p < 0.001* | *p < 0.001* |
|  |  |  |  |
| **Mean diastolic blood pressure (mmHg) (β)** | | | |
| **WC (cm)** |  |  |  |
| Q1 | 0.00 (Ref.) | 0.00 (Ref.) | 0.00 (Ref.) |
| Q2 | 2.83 (2.66, 3.00) <0.001 | 1.97 (1.79, 2.15) <0.001 | 2.01 (1.83, 2.19) <0.001 |
| Q3 | 5.09 (4.91, 5.26) <0.001 | 3.75 (3.57, 3.94) <0.001 | 3.79 (3.61, 3.98) <0.001 |
| Q4 | 7.89 (7.71, 8.07) <0.001 | 6.43 (6.23, 6.63) <0.001 | 6.46 (6.26, 6.65) <0.001 |
| *P for trend* | *p < 0.001* | *p < 0.001* | *p < 0.001* |
| **BMI (kg/m2)** |  |  |  |
| Q1 | 0.00 (Ref.) | 0.00 (Ref.) | 0.00 (Ref.) |
| Q2 | 2.11 (1.93, 2.29) <0.001 | 1.48 (1.30, 1.66) <0.001 | 1.45 (1.28, 1.63) <0.001 |
| Q3 | 4.26 (4.08, 4.43) <0.001 | 3.28 (3.10, 3.46) <0.001 | 3.25 (3.07, 3.42) <0.001 |
| Q4 | 7.24 (7.06, 7.42) <0.001 | 6.32 (6.14, 6.50) <0.001 | 6.26 (6.08, 6.44) <0.001 |
| *P for trend* | *p < 0.001* | *p < 0.001* | *p < 0.001* |
| **WHtR** |  |  |  |
| Q1 | 0.00 (Ref.) | 0.00 (Ref.) | 0.00 (Ref.) |
| Q2 | 3.03 (2.86, 3.21) <0.001 | 2.27 (2.09, 2.45) <0.001 | 2.32 (2.14, 2.50) <0.001 |
| Q3 | 5.15 (4.97, 5.33) <0.001 | 3.99 (3.80, 4.17) <0.001 | 4.06 (3.88, 4.24) <0.001 |
| Q4 | 7.16 (6.98, 7.34) <0.001 | 5.94 (5.75, 6.12) <0.001 | 6.01 (5.83, 6.20) <0.001 |
| *P for trend* | *p < 0.001* | *p < 0.001* | *p < 0.001* |
| **ABSI** |  |  |  |
| Q1 | 0.00 (Ref.) | 0.00 (Ref.) | 0.00 (Ref.) |
| Q2 | 2.36 (2.17, 2.54) <0.001 | 1.15 (0.96, 1.33) <0.001 | 1.23 (1.05, 1.41) <0.001 |
| Q3 | 3.28 (3.09, 3.46) <0.001 | 1.43 (1.24, 1.62) <0.001 | 1.58 (1.39, 1.77) <0.001 |
| Q4 | 3.09 (2.91, 3.28) <0.001 | 0.76 (0.57, 0.96) <0.001 | 0.99 (0.79, 1.18) <0.001 |
| *P for trend* | *p < 0.001* | *p < 0.001* | *p < 0.001* |
| **BRI** |  |  |  |
| Q1 | 0.00 (Ref.) | 0.00 (Ref.) | 0.00 (Ref.) |
| Q2 | 3.03 (2.86, 3.21) <0.001 | 2.27 (2.09, 2.45) <0.001 | 2.32 (2.14, 2.50) <0.001 |
| Q3 | 5.15 (4.97, 5.33) <0.001 | 3.99 (3.80, 4.17) <0.001 | 4.06 (3.88, 4.24) <0.001 |
| Q4 | 7.16 (6.98, 7.34) <0.001 | 5.94 (5.75, 6.12) <0.001 | 6.01 (5.83, 6.20) <0.001 |
| *P for trend* | *p < 0.001* | *p < 0.001* | *p < 0.001* |
|  |  |  |  |
| **Hypertension (OR)** | | | |
| **WC (cm)** |  |  |  |
| Q1 | 1.00 (Ref.) | 1.00 (Ref.) | 1.00 (Ref.) |
| Q2 | 1.95 (1.85, 2.05) <0.001 | 1.54 (1.46, 1.62) <0.001 | 1.55 (1.46, 1.63) <0.001 |
| Q3 | 2.91 (2.77, 3.05) <0.001 | 2.10 (2.00, 2.21) <0.001 | 2.11 (2.00, 2.23) <0.001 |
| Q4 | 4.44 (4.23, 4.66) <0.001 | 3.32 (3.15, 3.50) <0.001 | 3.33 (3.16, 3.51) <0.001 |
| *P for trend* | *p < 0.001* | *p < 0.001* | *p < 0.001* |
| **BMI (kg/m2)** |  |  |  |
| Q1 | 1.00 (Ref.) | 1.00 (Ref.) | 1.00 (Ref.) |
| Q2 | 1.53 (1.46, 1.61) <0.001 | 1.39 (1.32, 1.46) <0.001 | 1.37 (1.30, 1.45) <0.001 |
| Q3 | 2.13 (2.04, 2.24) <0.001 | 1.90 (1.80, 2.00) <0.001 | 1.87 (1.78, 1.97) <0.001 |
| Q4 | 3.28 (3.13, 3.43) <0.001 | 3.30 (3.14, 3.46) <0.001 | 3.22 (3.06, 3.38) <0.001 |
| *P for trend* | *p < 0.001* | *p < 0.001* | *p < 0.001* |
| **WHtR** |  |  |  |
| Q1 | 1.00 (Ref.) | 1.00 (Ref.) | 1.00 (Ref.) |
| Q2 | 2.23 (2.11, 2.36) <0.001 | 1.70 (1.60, 1.80) <0.001 | 1.71 (1.62, 1.81) <0.001 |
| Q3 | 3.43 (3.26, 3.62) <0.001 | 2.31 (2.18, 2.44) <0.001 | 2.34 (2.21, 2.47) <0.001 |
| Q4 | 5.88 (5.59, 6.19) <0.001 | 3.52 (3.33, 3.71) <0.001 | 3.57 (3.38, 3.77) <0.001 |
| *P for trend* | *p < 0.001* | *p < 0.001* | *p < 0.001* |
| **ABSI** |  |  |  |
| Q1 | 1.00 (Ref.) | 1.00 (Ref.) | 1.00 (Ref.) |
| Q2 | 1.75 (1.67, 1.84) <0.001 | 1.28 (1.21, 1.35) <0.001 | 1.30 (1.24, 1.37) <0.001 |
| Q3 | 2.28 (2.18, 2.39) <0.001 | 1.36 (1.29, 1.43) <0.001 | 1.41 (1.34, 1.48) <0.001 |
| Q4 | 2.98 (2.84, 3.12) <0.001 | 1.29 (1.22, 1.36) <0.001 | 1.36 (1.29, 1.43) <0.001 |
| *P for trend* | *p < 0.001* | *p < 0.001* | *p < 0.001* |
| **BRI** |  |  |  |
| Q1 | 1.00 (Ref.) | 1.00 (Ref.) | 1.00 (Ref.) |
| Q2 | 2.23 (2.11, 2.36) <0.001 | 1.70 (1.60, 1.80) <0.001 | 1.71 (1.62, 1.81) <0.001 |
| Q3 | 3.43 (3.26, 3.62) <0.001 | 2.31 (2.18, 2.44) <0.001 | 2.34 (2.21, 2.47) <0.001 |
| Q4 | 5.88 (5.59, 6.19) <0.001 | 3.52 (3.33, 3.71) <0.001 | 3.57 (3.38, 3.77) <0.001 |
| *P for trend* | *p < 0.001* | *p < 0.001* | *p < 0.001* |
| Notes: | | | |
| Model I: Unadjusted. | | | |
| Model II: Adjusted for age, gender, and ethnicity. | | | |
| Model III: Adjusted for age, gender, ethnicity, smoking status, family history of hypertension, history of diabetes, and history of coronary heart disease/myocardial infarction. | | | |
| Continuous outcomes (systolic blood pressure and diastolic blood pressure) are presented as β (95% CI). | | | |
| Binary outcome (hypertension) is presented as OR (95% CI). | | | |
| Q1 was used as the reference group for all analyses. | | | |
| P for trend was calculated by treating quartiles as continuous variables in regression models. | | | |

| **Table S3**  **Stratified analysis by gender.** | | | |
| --- | --- | --- | --- |
| **Exposure** | **Gender = male** | **Gender = female** | **Total** |
| **Hypertension (OR)** | | | |
| WC (cm) Z score | 1.64 (1.61, 1.68) | 1.50 (1.45, 1.54) | 1.64 (1.61, 1.66) |
|  | <0.001 | <0.001 | <0.001 |
| BMI (kg/m²) Z score | 1.64 (1.61, 1.68) | 1.51 (1.47, 1.55) | 1.63 (1.60, 1.66) |
|  | <0.001 | <0.001 | <0.001 |
| WHtR Z score | 1.67 (1.63, 1.71) | 1.46 (1.42, 1.50) | 1.60 (1.57, 1.63) |
|  | <0.001 | <0.001 | <0.001 |
| ABSI Z score | 1.09 (1.06, 1.11) | 1.04 (1.02, 1.07) | 1.11 (1.09, 1.13) |
|  | <0.001 | 0.002 | <0.001 |
| BRI Z score | 1.64 (1.60, 1.67) | 1.42 (1.38, 1.46) | 1.56 (1.53, 1.58) |
|  | <0.001 | <0.001 | <0.001 |
| **Mean systolic blood pressure (mmHg) (β)** | | | |
| WC (cm) Z score | 3.89 (3.76, 4.01) | 3.65 (3.49, 3.81) | 4.29 (4.20, 4.38) |
|  | <0.001 | <0.001 | <0.001 |
| BMI (kg/m²) Z score | 3.96 (3.84, 4.07) | 3.71 (3.57, 3.85) | 4.23 (4.14, 4.32) |
|  | <0.001 | <0.001 | <0.001 |
| WHtR Z score | 3.98 (3.85, 4.11) | 3.45 (3.30, 3.59) | 4.07 (3.98, 4.17) |
|  | <0.001 | <0.001 | <0.001 |
| ABSI Z score | 0.45 (0.31, 0.60) | 0.36 (0.22, 0.51) | 0.88 (0.78, 0.98) |
|  | <0.001 | <0.001 | <0.001 |
| BRI Z score | 3.98 (3.85, 4.11) | 3.39 (3.25, 3.53) | 4.03 (3.93, 4.13) |
|  | <0.001 | <0.001 | <0.001 |
| **Mean diastolic blood pressure (mmHg) (β)** | | | |
| WC (cm) Z score | 2.90 (2.81, 2.99) | 1.87 (1.77, 1.98) | 2.69 (2.62, 2.75) |
|  | <0.001 | <0.001 | <0.001 |
| BMI (kg/m²) Z score | 2.79 (2.71, 2.88) | 1.99 (1.90, 2.09) | 2.61 (2.55, 2.67) |
|  | <0.001 | <0.001 | <0.001 |
| WHtR Z score | 2.85 (2.76, 2.95) | 1.63 (1.53, 1.72) | 2.36 (2.29, 2.43) |
|  | <0.001 | <0.001 | <0.001 |
| ABSI Z score | 0.57 (0.46, 0.67) | -0.05 (-0.14, 0.05) | 0.45 (0.38, 0.52) |
|  | <0.001 | 0.320 | <0.001 |
| BRI Z score | 2.84 (2.75, 2.93) | 1.58 (1.49, 1.68) | 2.31 (2.25, 2.38) |
|  | <0.001 | <0.001 | <0.001 |
| Data in the table: β (95%CI) p-value/OR (95%CI) p-value. | | | |
| Outcome variables: Hypertension; Mean systolic blood pressure (mmHg); Mean diastolic blood pressure (mmHg). | | | |
| Exposure variables: ABSI Z score; Waist (cm) Z score; BMI (kg/m²) Z score; WHtR Z score; BRI Z score. | | | |
| Model adjusted for: Age; Race; Smoking status; Family history of hypertension; History of diabetes; History of CHD/MI. | | | |

| **Table S4**  **Stratified analysis by ethnicity.** | | | |
| --- | --- | --- | --- |
| **Exposure** | **Ethnicity = Han** | **Ethnicity = other** | **Total** |
| **Hypertension (OR)** | | | |
| WC (cm) Z score | 1.60 (1.57, 1.63) | 1.68 (1.60, 1.77) | 1.61 (1.58, 1.64) |
|  | <0.001 | <0.001 | <0.001 |
| BMI (kg/m²) Z score | 1.61 (1.58, 1.64) | 1.62 (1.55, 1.69) | 1.61 (1.58, 1.64) |
|  | <0.001 | <0.001 | <0.001 |
| WHtR Z score | 1.58 (1.56, 1.62) | 1.69 (1.62, 1.78) | 1.60 (1.57, 1.63) |
|  | <0.001 | <0.001 | <0.001 |
| ABSI Z score | 1.08 (1.06, 1.10) | 1.16 (1.10, 1.21) | 1.09 (1.07, 1.11) |
|  | <0.001 | <0.001 | <0.001 |
| BRI Z score | 1.55 (1.52, 1.58) | 1.65 (1.57, 1.72) | 1.56 (1.54, 1.59) |
|  | <0.001 | <0.001 | <0.001 |
| **Mean systolic blood pressure (mmHg) (β)** | | | |
| WC (cm) Z score | 3.99 (3.89, 4.10) | 3.95 (3.71, 4.19) | 3.99 (3.90, 4.09) |
|  | <0.001 | <0.001 | <0.001 |
| BMI (kg/m²) Z score | 4.02 (3.92, 4.12) | 3.89 (3.68, 4.11) | 4.01 (3.92, 4.10) |
|  | <0.001 | <0.001 | <0.001 |
| WHtR Z score | 3.93 (3.83, 4.03) | 4.03 (3.80, 4.26) | 3.96 (3.86, 4.05) |
|  | <0.001 | <0.001 | <0.001 |
| ABSI Z score | 0.60 (0.49, 0.71) | 0.61 (0.35, 0.86) | 0.61 (0.51, 0.71) |
|  | <0.001 | <0.001 | <0.001 |
| BRI Z score | 3.90 (3.80, 4.00) | 4.04 (3.81, 4.27) | 3.93 (3.84, 4.03) |
|  | <0.001 | <0.001 | <0.001 |
| **Mean diastolic blood pressure (mmHg) (β)** | | | |
| WC (cm) Z score | 2.43 (2.36, 2.51) | 2.67 (2.50, 2.85) | 2.48 (2.41, 2.54) |
|  | <0.001 | <0.001 | <0.001 |
| BMI (kg/m²) Z score | 2.44 (2.37, 2.51) | 2.54 (2.39, 2.70) | 2.46 (2.39, 2.52) |
|  | <0.001 | <0.001 | <0.001 |
| WHtR Z score | 2.22 (2.15, 2.30) | 2.58 (2.41, 2.75) | 2.28 (2.22, 2.35) |
|  | <0.001 | <0.001 | <0.001 |
| ABSI Z score | 0.23 (0.15, 0.31) | 0.45 (0.27, 0.64) | 0.27 (0.20, 0.34) |
|  | <0.001 | <0.001 | <0.001 |
| BRI Z score | 2.18 (2.11, 2.26) | 2.57 (2.40, 2.74) | 2.25 (2.18, 2.31) |
|  | <0.001 | <0.001 | <0.001 |
| Data in the table: β (95%CI) p-value/OR (95%CI) p-value. | | | |
| Outcome variables: Hypertension; Mean systolic blood pressure (mmHg); Mean diastolic blood pressure (mmHg). | | | |
| Exposure variables: ABSI Z score; Waist (cm) Z score; BMI (kg/m²) Z score; WHtR Z score; BRI Z score. | | | |
| Model adjusted for: Age; Gender; Smoking status; Family history of hypertension; History of diabetes; History of CHD/MI. | | | |

| **Table S5** | | | |
| --- | --- | --- | --- |
| **Index** | **β （95%CI）** | **P-value** | **Direction** |
| WC | -0.013[-0.0142, -0.0119] | < 0.001 | Negative |
| BMI | -0.0075[-0.0086, -0.0065] | < 0.001 | Negative |
| WHtR | -0.0147[-0.0158, -0.0135] | < 0.001 | Negative |
| ABSI | -0.0091[-0.0102, -0.0080] | < 0.001 | Negative |
| BRI | -0.0141[-0.0152, -0.0131] | < 0.001 | Negative |
| Note:  BMI, body mass index; WC, waist circumference; WHtR, waist-to-height ratio; ABSI, a body shape index; BRI, body roundness index; β, regression coefficient; CI, confidence interval. The coefficient represents the per-unit change associated with a 1-unit increase in the Z-score of each index. All reported associations are statistically significant (P < 0.001) and indicate a negative direction. | | | |

| **Table S6**  **Multivariate regression analysis.** | | | |
| --- | --- | --- | --- |
| **Exposure** | **Model I** | **Model II** | **Model III** |
| **Hypertension (OR)** | | | |
| WC (cm) Z score | 1.73 (1.70, 1.76) | 1.61 (1.58, 1.64) | 1.61 (1.58, 1.64) |
|  | <0.001 | <0.001 | <0.001 |
| BMI (kg/m²) Z score | 1.54 (1.52, 1.57) | 1.62 (1.60, 1.65) | 1.61 (1.58, 1.64) |
|  | <0.001 | <0.001 | <0.001 |
| WHtR Z score | 1.88 (1.85, 1.91) | 1.59 (1.56, 1.62) | 1.60 (1.57, 1.63) |
|  | <0.001 | <0.001 | <0.001 |
| ABSI Z score | 1.48 (1.45, 1.50) | 1.07 (1.05, 1.09) | 1.09 (1.07, 1.11) |
|  | <0.001 | <0.001 | <0.001 |
| BRI Z score | 1.82 (1.79, 1.85) | 1.55 (1.53, 1.58) | 1.56 (1.54, 1.59) |
|  | <0.001 | <0.001 | <0.001 |
| **Mean systolic blood pressure (mmHg) (β)** | | | |
| WC (cm) Z score | 5.34 (5.25, 5.44) | 3.98 (3.88, 4.07) | 3.99 (3.89, 4.09) |
|  | <0.001 | <0.001 | <0.001 |
| BMI (kg/m²) Z score | 4.60 (4.51, 4.70) | 4.05 (3.96, 4.14) | 4.00 (3.91, 4.09) |
|  | <0.001 | <0.001 | <0.001 |
| WHtR Z score | 5.84 (5.75, 5.94) | 3.92 (3.83, 4.02) | 3.95 (3.86, 4.05) |
|  | <0.001 | <0.001 | <0.001 |
| ABSI Z score | 3.39 (3.29, 3.49) | 0.49 (0.39, 0.59) | 0.61 (0.51, 0.71) |
|  | <0.001 | <0.001 | <0.001 |
| BRI Z score | 5.78 (5.68, 5.87) | 3.90 (3.81, 4.00) | 3.93 (3.83, 4.02) |
|  | <0.001 | <0.001 | <0.001 |
| **Mean diastolic blood pressure (mmHg) (β)** | | | |
| WC (cm) Z score | 2.98 (2.91, 3.04) | 2.46 (2.39, 2.53) | 2.47 (2.41, 2.54) |
|  | <0.001 | <0.001 | <0.001 |
| BMI (kg/m²) Z score | 2.78 (2.71, 2.84) | 2.48 (2.42, 2.54) | 2.46 (2.39, 2.52) |
|  | <0.001 | <0.001 | <0.001 |
| WHtR Z score | 2.68 (2.61, 2.74) | 2.25 (2.19, 2.32) | 2.28 (2.22, 2.35) |
|  | <0.001 | <0.001 | <0.001 |
| ABSI Z score | 1.07 (1.01, 1.14) | 0.18 (0.11, 0.25) | 0.27 (0.20, 0.34) |
|  | <0.001 | <0.001 | <0.001 |
| BRI Z score | 2.63 (2.57, 2.69) | 2.22 (2.15, 2.28) | 2.25 (2.18, 2.31) |
|  | <0.001 | <0.001 | <0.001 |
| Data in the table: β (95%CI) p-value/OR (95%CI) p-value. | | | |
| Outcome variables: Hypertension; Mean systolic blood pressure (mmHg); Mean diastolic blood pressure (mmHg). | | | |
| Exposure variables: ABSI Z score; Waist (cm) Z score; BMI (kg/m²) Z score; WHtR Z score; BRI Z score. | | | |
| Model I adjust for: None. | | | |
| Model II, adjust for: Gender; Age (years); Race. | | | |
| Model III, adjust for: Gender; Age (years); Race; Smoking status; Family history of hypertension; History of diabetes; History of CHD/MI. | | | |

Figure 1.

Flowchart of study population selection.

Figure 2.

Receiver operating characteristic (ROC) curves for discriminating hypertension in the overall population.

Detailed results of univariate, multivariable, and stratified analyses are provided in the Supplementary Material (Tables S1-S6).

Figure 3

ROC curves for discriminating hypertension, stratified by age. (A) Youth group, (B) Middle-aged group, (C) Elderly group.

Figure 4

ROC curves for discriminating hypertension in the youth population, stratified by ethnicity.

(A)Han youth group, (B) Other Ethnicity youth group.

Figure 5

ROC curves for discriminating hypertension in the youth population, stratified by sex.

(A) Youth male group, (B) Youth female group.
